# How to Use Respiratory Chain Inhibitors in Toxicology Studies—Whole-Cell Measurements

**DOI:** 10.3390/ijms23169076

**Published:** 2022-08-13

**Authors:** Mariusz Żuberek, Patrycja Paciorek, Michał Rakowski, Agnieszka Grzelak

**Affiliations:** 1 Cytometry Laboratory, Department of Molecular Biophysics, Faculty of Biology and Environmental Protection, University of Lodz, 90-236 Lodz, Poland; 2 The Bio-Med-Chem Doctoral School of the University of Lodz and Lodz Institutes of the Polish Academy of Sciences, University of Lodz, 90-237 Lodz, Poland

**Keywords:** electron transport chain inhibition, rotenone, antimycin A, reactive oxygen species, mitochondrial membrane potential

## Abstract

Mitochondrial electron transport chain (ETC) inhibition is a phenomenon interesting in itself and serves as a tool for studying various cellular processes. Despite the fact that searching the term “rotenone” in PubMed returns more than 6900 results, there are many discrepancies regarding the directions of changes reported to be caused by this RTC inhibitor in the delicate redox balance of the cell. Here, we performed a multifaceted study of the popular ETC inhibitors rotenone and antimycin A, involving assessment of mitochondrial membrane potential and the production of hydrogen peroxide and superoxide anions at cellular and mitochondrial levels over a wide range of inhibitor concentrations (1 nmol/dm^3^–100 µmol/dm^3^). All measurements were performed with whole cells, with accompanying control of ATP levels. Antimycin A was more potent in hindering HepG2 cells’ abilities to produce ATP, decreasing ATP levels even at a 1 nmol/dm^3^ concentration, while in the case of rotenone, a 10,000-times greater concentration was needed to produce a statistically significant decrease. The amount of hydrogen peroxide produced in the course of antimycin A biological activity increased rapidly at low concentrations and decreased below control level at a high concentration of 100 µmol/dm^3^. While both inhibitors influenced cellular superoxide anion production in a comparable manner, rotenone caused a greater increase in mitochondrial superoxide anions compared to a modest impact for antimycin A. IC50 values for rotenone and antimycin A with respect to HepG2 cell survival were of the same order of magnitude, but the survival curve of cells treated with rotenone was clearly biphasic, suggesting a concentration-dependent mode of biological action. We propose a clear experimental setup allowing for complete and credible analysis of the redox state of cells under stress conditions which allows for better understanding of the effects of ETC inhibition.

## 1. Introduction

Aerobic organisms are permanently in danger of oxidative stress (OS). OS is a metabolic state of the cell that occurs when the balance between reactive oxygen species (ROS) and the antioxidant defense system is disturbed, resulting in excessive amounts of reactive oxygen species. As a result, cell metabolism is disturbed by deregulation of intracellular signaling pathways and damage to essential cell building elements, such as proteins, lipids and nucleic acids, occurs [1].

OS is considered an important factor associated with many pathological conditions, such as cancer [2], neurodegenerative [3], cardiovascular [4] and immunological background diseases [5].

In some cases, OS is believed to underlie the etiology of a disease and in others to be an important component of the outcome of a disease. Many research papers have focused on the development of means to and the search for substances that restore the normal redox state in cells, which in turn can eliminate pathological conditions or promote the efficacy of conventional therapies [5].

ROS, including oxygen free radicals, present in cells in the normal, physiological state, are essential for the proper functioning of cells and whole organisms. They are also produced in response to physical, chemical and biological factors that can cause cell damage. Intracellular signaling pathways that are activated by free radicals include NF-κB and AP1 [6]. In the cell, oxygen free radicals are produced mainly by NADPH oxidases [7] and in side reactions of the mitochondrial electron transport chain [8]. The mitochondrial electron transport chain is a structure composed of interrelated protein complexes located in the inner mitochondrial membrane which under physiological conditions is responsible for carrying out oxidative phosphorylation reactions. ETC was first identified as a site for the production of ROS in 1966 [9].

In eukaryotic organisms, oxidative phosphorylation involves oxidoreductases defined as mitochondrial complexes. Initially, it was assumed that mitochondrial complexes are randomly distributed in the inner mitochondrial membrane, but now it is accepted that they are organized in supercomplexes [10]. Such configurations are surely optimal in regard to electron transfer between the involved carriers and oxidoreductases [11]. Mitochondrial studies are often conducted on isolated mitochondria. Such experiments have established that mitochondrial metabolism is the main source of reactive oxygen species in the cell [12,13,14]. At the initial stage of research on mitochondrial metabolism, hydrogen peroxide was identified as the main by-product of mitochondrial chain activity. The discovery that mitochondria are capable of superoxide anion dismutation via autonomous MnSOD allowed the positive verification of the hypothesis that mitochondria produce superoxide anions [15,16].

Mitochondrial superoxide anions are produced by the single-electron reduction of O_2_, and it is estimated that intramitochondrial concentrations of superoxide anions in vivo settle at a level ranging from 3 µmol/dm^3^ to 30 µmol/dm^3^ [17]. At the organismal level, the average superoxide anion concentration is estimated to be in the range of 10 to 200 pmol/dm^3^ [8,12].

The wide range of concentrations reported is due to the fact that it is very difficult to determine superoxide concentration under in vivo conditions. This difficulty is in part due to the presence of large amounts of superoxide dismutases (SODs). Large amounts of SODs are present in mitochondria (the matrix of rat liver mitochondria contains MnSOD at a concentration of 11 µmol/dm^3^) [18], and the reaction of superoxide dismutation is characterized by a very high rate constant. It is worth noting that there are studies that have reported the existence of CuZnSOD in the mitochondrial intermembrane space as well [19]. Many mitochondrial proteins have the potential to facilitate superoxide anion formation, which has been experimentally proven for isolated proteins, although extrapolation of such data to in vivo systems is very limited.

It is crucial to understand how superoxide anions are produced in mitochondria in vivo and what the limitations of experimental systems are in terms of prediction of cellular and organismal response to oxidative stress based on isolated mitochondria.

**Figure 1:**
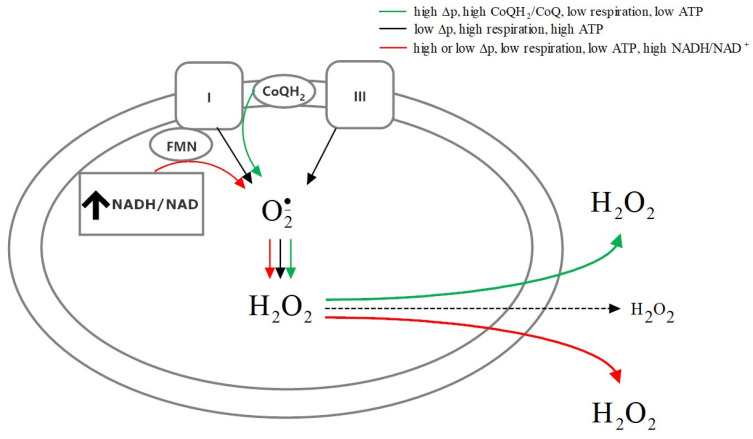
The functional state of mitochondria governs the mechanism of reactive oxygen species production. Mitochondrial production of superoxide radicals is higher when ATP synthesis in the course of oxidative phosphorylation is low coupled with a high ratio of NADH/NAD^+^ or CoQH_2_/CoQ [20]. The active mitochondrial electron transport chain promotes induction of cellular antioxidant defense mechanisms [21], decreasing the amount of detectable ROS.

The key factors determining mitochondrial ROS production are mitochondrial membrane potential and intracellular ATP and NADH levels available for oxidative phosphorylation. Murphy [20] proposed three conditions of mitochondrial activity leading to the generation of high amounts of superoxide, which are summarized in Figure 1.

Mitochondrial complexes I and III were identified as the main sources of superoxide radicals in mitochondria. Complex I is a large protein complex with a total molecular mass of around 1 MDa composed of 45 polypeptides [22,23,24,25]. It produces large amounts of $O_{2}^{\dot{-}}$ and was initially considered to be crucial to the production of ROS in mitochondria. When the NADH/NAD^+^ ratio is high, the rate of electron transfer between NADH and FMN is increased and consequently the production of superoxide anions is elevated. The reverse electron transfer mechanism of superoxide anion generation has been proposed under conditions of elevated mitochondrial membrane potential and high CoQH_2_/CoQ ratios [26,27,28]. Complex III contains heme groups in cytochrome c_1_ and cytochrome b and a sulphur–iron center in Rieske protein and is capable of transient interaction with CoQ during the Q-cycle at the Qi and Qo sites [29]. Complex III has long been considered a source of superoxide anions secreted into the mitochondria [30,31,32,33]. Eleven mitochondrial sites involved in substrate oxidation and oxidative phosphorylation leak electrons to oxygen, producing superoxide or hydrogen peroxide–oxoacid dehydrogenase complexes that feed electrons to NAD+, respiratory complexes I and III, and dehydrogenases, including complex II, which uses ubiquinone as an acceptor. Complex III and mitochondrial glycerol 3-phosphate dehydrogenase release superoxide into the intermembrane space and into the matrix, while the other sites generate superoxide and/or hydrogen peroxide exclusively in the matrix. Sites I_F_, III_Qo_ and G_Q_ are thought to generate superoxide predominantly or exclusively. Site II_F_ may generate both superoxide and hydrogen peroxide. The electron leak products of sites O_F_, P_F_, B_F_, A_F_, I_Q_, E_F_ and D_Q_ may be mostly superoxide or a mixture of superoxide and hydrogen peroxide [34].

**Figure 2:**
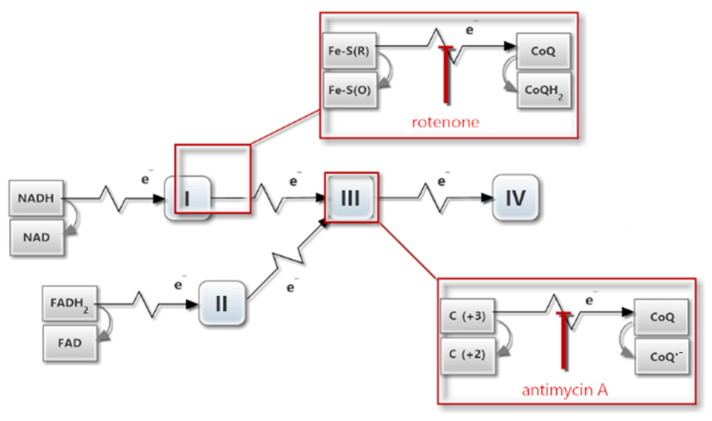
Key sites of mitochondrial electron transport chain inhibition by rotenone and antimycin A. NAD (Nicotinamide adenine dinucleotide), FAD (flavin adenine dinucleotide), Fe-S (Iron–sulfur clusters; R−reduced, O−oxidized), CoQ (Coenzyme Q, ubiquinone), CoQ·^−^ (semiquinone radical anion), C(+2) and C(+3) (Cytochrome b Fe^II^ and Cytochrome b Fe^III^, respectively). The numbers I–IV symbolize structural complexes in the respiratory chain.

Inhibition of the Qi site with antimycin A (Figure 2), while the level of CoQH_2_ is high, results in the production of large amounts of superoxide anions, which are detectable both in the mitochondrial matrix and in the intermembrane space [35,36,37,38].

A key factor in the studies on mitochondrial activity has been the use of selective inhibitors of the respiratory chain. The most commonly used and best studied in terms of their biological effects are rotenone and antimycin A. Although inhibition by these agents is not complete, they are very efficient and useful in this area of research.

Rotenone is an isoflavone that is naturally produced by plants and has the ability to inhibit complex I [39,40]. Rotenone is a cell death-inducing agent, the efficacy of which has been confirmed in many cell lines of different tissue origin [41,42,43,44,45,46].

It has been found that oxygen free radicals generated in the course of respiratory chain inhibition by rotenone can significantly affect the condition of mitochondria themselves, although they are not capable of affecting other cellular structures, as they are not able to diffuse beyond the mitochondria [30].

Antimycin A is a secondary metabolite produced by *Streptomyces Kitazawensis* [47], which is used as an inhibitor of complex III. After administration of antimycin A, electron flow through the mitochondrial chain is disrupted, resulting in the derangement of mitochondrial membrane potential [48,49,50], increased production of oxygen free radicals [48,51] and decrease in intracellular ATP concentration [49,52].

In most cases, the measurement of reactive oxygen species levels is conducted with fluorescent probes, including derivatives of rhodamine, ethidine and fluorescein. Ethidine and its cationic derivatives are used for the detection of superoxide anions. An ethidine derivative, MitoSOX, is modified in a way that promotes its accumulation in mitochondria; hence, it is applicable for measurement of superoxide anions in the mitochondrial matrix. The oxidation of MitoSOX by superoxide radicals leads to its hydroxylation at the 2-position, and analysis of the oxidation product fluorescence spectrum allows the determination of whether the probe was oxidized by superoxide anions or by other ROS [53].

Within the cytoplasm, dihydroetidine (DHE) exhibits weak blue fluorescence, and after oxidation to ethidine intercalates to DNA and shows intense red fluorescence. DHE has been used for the measurement of the production of mitochondrial superoxide radicals [54], although MitoSOX is characterized by a much greater specificity because it is located directly in the mitochondria. Both of these dyes are used to evaluate the level of generation of superoxide anions in different compartments of the cell [55].

A relatively selective fluorescent probe for determining the generation of reactive oxygen species is dihydrorhodamine 123. It is a non-charged and non-fluorescent, reduced form of rhodamine 123 which has the ability to penetrate biological membranes. This dye does not directly react with superoxide anions, but exhibits reactivity for peroxides, especially in the presence of peroxidases, cytochrome c or Fe^2+^ [56]. It can also react with peroxynitrite [57].

Mitochondrial membrane potential is a parameter that allows evaluation of the functional state of mitochondria. It is estimated mainly using fluorometric probes based on fluorone and carbocyanine dye families. The most widely used mitochondrial membrane potential probes are JC-1 and JC-9. Both probes are ratiometric; the change in the monomer-to-dimer fluorescence ratio is a measure of mitochondrial membrane potential.

A different approach to estimate mitochondrial potential fluorometrically employs the probe CMXRos, which does not undergo any modifications in the cell, the level of its accumulation in mitochondria depending only on the polarization of the inner mitochondrial membrane.

The estimation of ROS production using isolated mitochondria is prone to artifact interference due to the possible damage of these organelles during the isolation procedure and lack of interaction with the cellular environment.

This work aims to explain the discrepancies between results reported in the literature concerning the influence of mitochondrial electron transport chain inhibitors on cellular redox balance and to propose an accurate method by means of which to estimate the levels of reactive oxygen species in intact cellular systems. The probes used in this study allow for evaluation of the levels of reactive oxygen species in respective cellular compartments. Measurement of ROS, mitochondrial membrane potential and ATP content across a wide range of concentrations of commonly used inhibitors allows for the precise determination of the kinetics of ROS production by active mitochondria in cellular systems.

## 2. Results

### 2.1. Survival of HepG2 Cells after Treatment with Rotenone and Antimycin A

**Figure 3:**
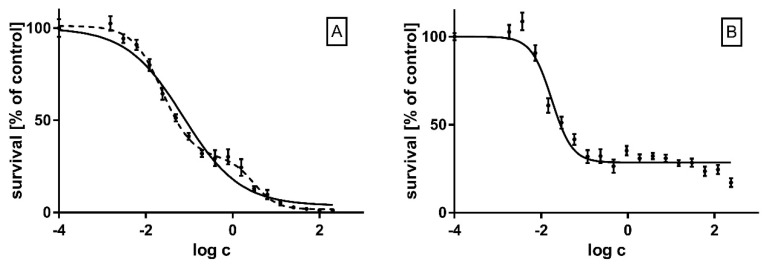
Survival of HepG2 cells after 24 h treatment with increasing concentrations of rotenone (**A**) and antimycin A (**B**). Inhibitors were tested at concentrations ranging from 1.5 nmol/dm^3^ and 1.8 nmol/dm^3^ to 200 µmol/dm^3^ and 240 µmol/dm^3^ for rotenone and antimycin A, respectively. Decreasing concentrations of the inhibitors were obtained by subsequent double dilutions. Assays were performed in six independent repetitions. EC50 values: rotenone—56.15 nmol/dm^3^ (95%CI: 40.55 to 77.78 nmol/dm^3^), antimycin A—15.97 nmol/dm^3^ (95%CI: 13.23 to 19.03 nmol/dm^3^).

Survival of HepG2 cells after treatment with rotenone and antimycin was assessed 24 h after treatment, a time which corresponds to the acute toxicity of the studied compounds. IC50 values for rotenone and antimycin A were found to be 56.15 nmol/dm^3^ and 15.97 nmol/dm^3^, respectively. However, the dependence of survival on the concentration of rotenone is clearly biphasic. Rotenone is a compound characterized by a broad spectrum of biological activity which does not only inhibit complex I of the respiratory chain, but also affects other processes crucial for cell redox balance, which apparently accounts for the biphasicity of its dose–effect curve (Figure 3). In the measurement of reactive oxygen species, mitochondrial membrane potential and ATP level, we used concentrations and incubation times in which no significant toxic effect was observed while at the same time the biological effects of the inhibitors could be observed.

### 2.2. Production of Reactive Oxygen Species

**Figure 4:**
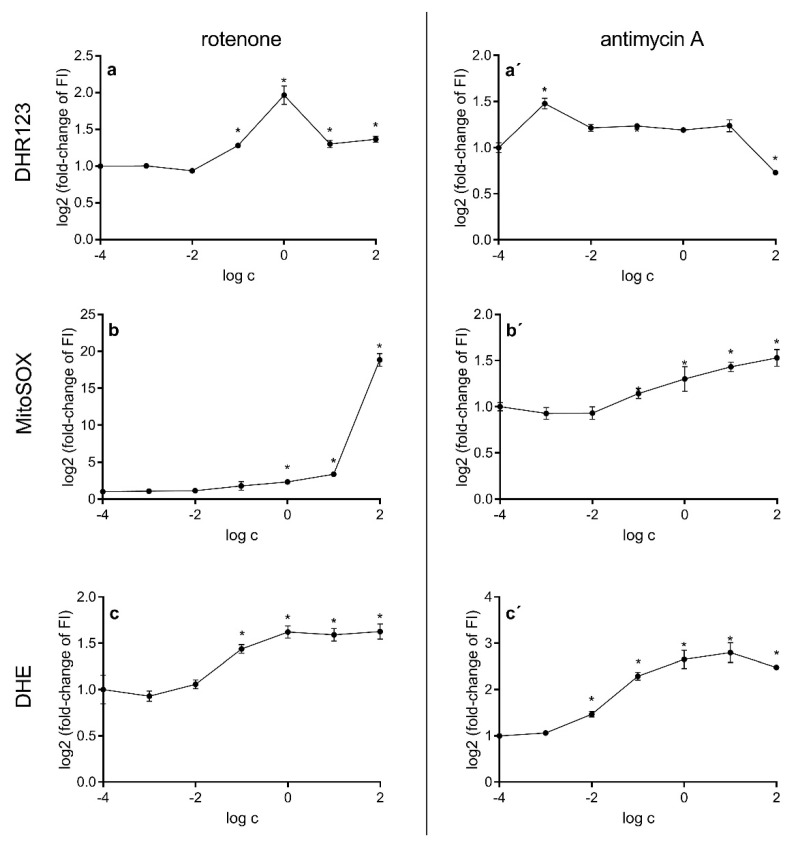
Production of reactive oxygen species in HepG2 cells after treatment with rotenone (**a**,**b**,**c**) and antimycin A (**a**’,**b**’,**c**’). Hydrogen peroxide (**a**,**a**’), mitochondrial superoxide radicals (**b**,**b**’) and cellular superoxide radicals (**c**,**c**’) were measured with dihydrorhodamine 123, MitoSOX and dihydroethidium, respectively. Measurements were performed after 4 h of incubation with rotenone or antimycin A. Asterisks denote statistically significant changes (analysis of variance, Dunnett’s post hoc test, *n* = 3, α = 0.05).

Incubation of HepG2 cells with both mitochondrial electron transport chain inhibitors, antimycin A and rotenone (Figure 4), resulted in concentration-dependent changes in the production of reactive oxygen species. In both cases, the use of inhibitors induced an increase in the production of hydrogen peroxide and superoxide radicals on the mitochondrial and cellular levels. However, antimycin A did not cause an increase in the generation of hydrogen peroxide at all studied concentrations, the highest of which actually caused a decrease in this parameter. Another difference between the inhibitors worth mentioning is the amount of superoxide radicals produced after treatment with a high concentration of rotenone, a 19-fold increase being reached with respect to control cells.

### 2.3. Mitochondrial Membrane Potential

**Figure 5:**
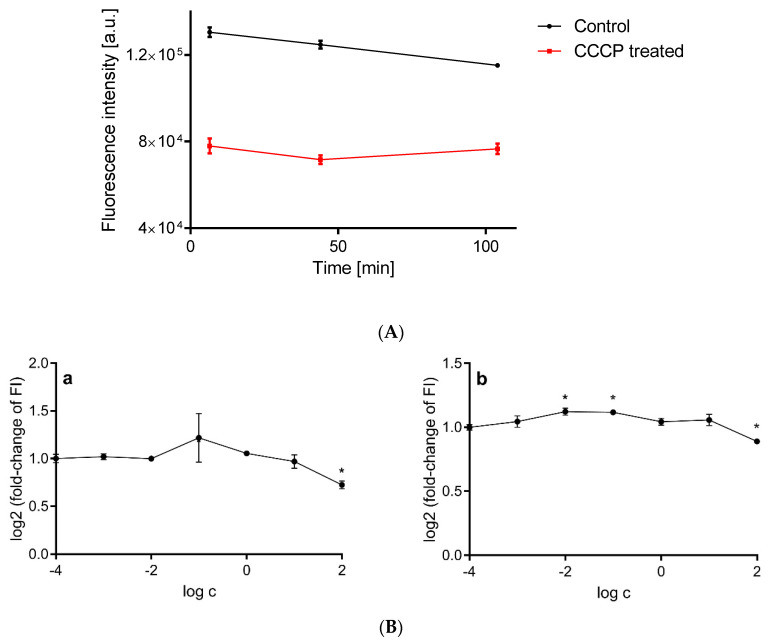
(**A**) Measurement of mitochondrial potential in HepG2 cells treated with CCCP (carbonyl cyanide m-chlorophenylhydrazone) at a concentration of 5 μmol/dm^3^. (**B**) Mitochondrial membrane potential of HepG2 cells after 4 h treatment with rotenone (**a**) and antimycin A (**b**), expressed as fluorescence of CMXRos probe. Asterisks denote statistically significant changes towards control (analysis of variance, Dunnett’s post hoc test, *n* = 3, α = 0.05).

To validate the measurement of mitochondrial potential with CMXRos, we used CCCP, which is a recognized mitochondrial uncoupler that causes dissipation of mitochondrial membrane potential [58]. CCCP in effect lowers the fluorescence readout for CMXRos (Figure 5A), which suggests that the measurement with this dye is an effective tool with which to determine the mitochondrial potential in the presented test system.

Both studied inhibitors decreased the mitochondrial membrane potential in HepG2 cells after 4 h of incubation, but only at a concentration of 100 µmol/dm^3^. Interestingly, antimycin A increased the mitochondrial membrane potential at submicromolar concentrations of 1 and 10 nmol/dm^3^ (Figure 5B).

### 2.4. ATP Concentration

**Figure 6:**
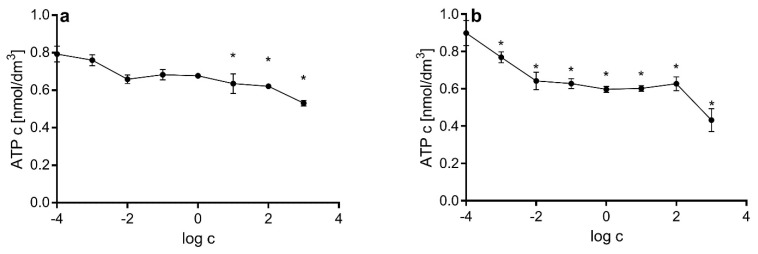
ATP concentration in HepG2 cells after 4 h treatment with rotenone (**a**) and antimycin A (**b**), expressed in relation to protein concentration. Asterisks denote statistically significant changes towards control (analysis of variance, Dunnett’s post hoc test, *n* = 3, α = 0.05).

ATP concentration was assessed in HerG2 cells after 4 h of incubation with electron transport chain inhibitors. Both inhibitors caused a concentration-dependent decrease in cellular ATP concentration. Statistically significant changes were detected with lower concentrations of antimycin A (1 nmol/dm^3^) relative to rotenone (10 µmol/dm^3^) (Figure 6).

## 3. Discussion

As described before [21], HepG2 cells respond differently to oxidative stress depending on the concentration of glucose in the culture medium. This effect is tied to the dynamic balance between the glycolysis process and the activity of the mitochondrial respiratory chain. Despite ATCC giving clear recommendations concerning conditions for HepG2 cell line culture, including the recommendation of a glucose concentration of 5.5 mmol/dm^3^, there is a wide range of culture media and glucose concentrations used across different studies. In fact, in most studies in which the HepG2 cell line is used, culture methods employ media with much higher glucose concentrations, e.g., RPMI-1640 (11 mmol/dm^3^) [59], DMEM (25 mmol/dm^3^) [60] or there are no data on which medium (or which formulation variant of a given medium) was used for the growth of the cells [61,62,63,64]. Dulbecco’s modified Eagle medium formulation is perhaps the most variable, and virtually no researchers state which formulation variant they use in their studies. Thermo Fisher Scientific alone offers 15 formulations of DMEM. As exogenous glucose concentration has a crucial regulatory effect on cell energy metabolism, lack of information on the glucose level of the medium used can seriously hinder the interpretation of results. It is also of note that a glucose concentration of 5.5 mmol/dm^3^ corresponds to the proper physiological state, whereas a concentration of 25 mmol/dm^3^ used in many commercially available media corresponds to acute diabetes shock. In this paper, we used a medium with an elevated glucose concentration (DMEM, 25 mmol/dm^3^) to facilitate the comparison of the presented results with those of other papers on the subject. The differences in response to oxidative and nitrogen stress for different media were significant and have been analyzed in our previous papers [21,65]. Aside from glucose, other compounds included in the formulations of culture media can complicate inferences, especially considering studies on cellular redox balance, as HEPES was found to modulate ATP production [66] and phenol red was shown to facilitate the generation of ROS [67].

For the determination of cell survival, neutral red was used as an assay with a relatively low dependence on the redox environment of the cell. Despite the impossibility of achieving complete independence of the assay from oxidation and reduction reactions in the cell environment modulated with xenobiotics, neutral red seems to be a better solution compared with the tests based on the reduction of tetrazolium dyes. It has been shown that the effects of the popular MTT-based assays depend on the metabolic states of cells, in particular, on the activity of NAD(P)H-dependent oxidoreductases [68]. Neutral red staining is not without its drawbacks. The assay itself is based on the accumulation of the dye in the lysosomes of living cells. Dye molecules at cellular physiological pH do not have a charge, while after penetrating into lysosome environments of lower pH, they become charged. The charge traps neutral red in lysosomes by preventing transmembrane diffusion. If the cell’s metabolism cannot maintain an adequate pH gradient, the assay reading may be distorted [69]. At the same time, it was previously shown that the neutral red assay produced similar results to the lactate dehydrogenase leakage assay in HepG2 cells treated with the mitochondria disruption agent cadmium chloride [70].

There is a vast disparity between the rotenone concentrations used, not only across unrelated cell lines, but also among cell lines of similar type [71,72]. Rotenone is widely used to induce neurodegenerative disorders in test animals and is considered to evoke a stable and reproductive model of Parkinson’s disease [73]. The neurodegenerative properties of rotenone have often been associated with its potential to inhibit the activity of electron transport chains. However, test animals lacking the Ndufs4 gene coding for a vital subunit of Complex I did not present Parkinson’s disease symptoms, nor did Complex I dysfunction cause neuronal death [74]. Apart from the inhibitory activity of rotenone, this compound does present a range of other mechanisms of biological activity mediating cell death, such as microtubule depolarization, oxidative stress and, in the case of dopamine neurons, accumulation of cytosol dopamine, which conditions the increased sensitivity of these cells to rotenone [74].

The inhibitory action of rotenone towards microtubule polarization has been confirmed across a variety of cell lines, including HeLa and MCF-7. Even low concentrations of rotenone (0.2–0.4 µmol/dm^3^) hindered the development of microtubules, influenced the rate of GTP hydrolysis, caused a decrease in the spaces between centrosomes and promoted the formation of multipolar spindles, which led to multipolar mitosis [72].

Changes in centrosome organization have been widely studied, as they are tied to aneuploidy, which plays an important role in inflammation and carcinogenesis [75,76]. Disturbances in the regulation of the centrosome division cycle or lack of coordination between centrosomes and the cell cycle leads to the instability of chromosomes and changes in their numbers [77]. On the other hand, the development of multipolar spindle apparatuses can be also harmful to cancer cells. While three-polar cells still undergo cytokinesis, higher number of poles inhibit this process. Moreover, it was found that rotenone influences the rate of GTP hydrolysis.

There are some quite contradictory works assessing the actual ability of rotenone to promote the production of ROS. Some papers have described the increase in cytoplasmic levels of ROS in response to rotenone [41,78], while others have reported opposite results [79,80,81].

The key to understanding this discrepancy is the analysis of this parameter in relation to other parameters crucial to cell metabolism, i.e., ATP level, mitochondrial membrane potential and other parameters influencing the intracellular distribution of ROS produced as a consequence of mitochondria dysfunction.

Apart from the promotion of ROS production by rotenone due to electron transport chain blockade, this compound also induces the expression of enzymes heavily contributing to the ROS pool in cells, such as microglial phagocyte NADPH oxidase (PHOX). Rotenone directly binds to the membrane-bound gp91 subunit activating the enzyme. Rotenone also binds to the RAC 1 protein, which can also lead to increase in PHOX activity [82].

The very high potential of rotenone to induce production of mitochondrial superoxide radicals in comparison to antimycin A (Figure 4) is not entirely related to its capability to inhibit the mitochondrial electron transport chain. The influence of rotenone on microtubule polymerization is closely tied to mitochondrial membrane potential. As another study has shown [72], rotenone effectively blocks the assembly of microtubules, which in turn have been shown to reduce mitochondrial membrane potential [83]. Lowered mitochondrial membrane potential (Figure 5), together with decreased ATP concentration (Figure 6), leads to rapid increase in the production of superoxide radicals (Figure 2). The proposed cause-and-effect relationship is typical for high concentrations of rotenone. It is also of note that tubulin is a crucial component of the mitochondrial membrane and promotes closure of VDAC. Thus, we postulate that the equilibrium of rotenone cytotoxic activity is shifted to a free radical mechanism at lower concentrations and to a tubulin-binding mechanism at higher concentrations. The relatively small effect of respiratory chain inhibitors on ATP levels (Figure 6) is probably due to the compensation of ATP loss by the glycolytic pathway. It has been shown previously that cancer cells obtain energy mainly through anaerobic glycolysis, the so-called Warburg effect [84]. Cell cultivation carried out in media with a high glucose content promotes this phenomenon. It has also been shown that cells using glycolysis can efficiently maintain the necessary potential of the mitochondrial membrane, as is the case with macrophages [85]. In the case of the experimental system used in this study, namely, HepG2 cells, the use of inhibitors moderately reduced ATP levels and caused a decrease in mitochondrial membrane potential only at high concentrations of electron transport chain inhibitors (Figure 5B). Compensation was possible thanks to the reverse activity of ATP synthase, which, utilizing the ATP generated in glycolysis, can stabilize the mitochondrial membrane potential and the proton gradient [86]. It is highly probable that this is also the case in other studies where high-glucose media are used for the culture of cells that use anaerobic glycolysis as an energy source.

The biphasic nature of the function describing the survival of HepG2 cells subjected to rotenone treatment suggests that rotenone, in modest concentrations, up to 0.01 µmol/dm^3^, promotes the development of adaption mechanisms and that after crossing the 1.5 µmol/dm^3^ threshold, the defense mechanisms against oxidative stress are overwhelmed such that a rapid decline in cell survival is observed. Another explanation of such a peculiar survival curve is the possibility that, at low concentrations, rotenone inhibits oxidative phosphorylation, yet, although it does hinder cell metabolism, cell survival is still possible due to glycolysis, while high concentrations disturb microtubule metabolism and this effect cannot be compensated, resulting in total cell death.

The second explanation is supported by the results of studies on the survival of HepG2 cells exposed to antimycin A. Antimycin A does not have any other mechanisms of cytotoxic action other than ETC inhibition. In our experiments, we were unable to achieve a total loss of survival of HepG2 cells under antimycin A treatment, although the maximal concentrations used ranged up to 200 µmol/dm^3^.

After complex analysis of previously mentioned parameters over a wide range of concentrations of the studied inhibitors, we can state that antimycin A, at low concentrations ranging from 1 nmol/dm^3^ to 10 nmol/dm^3^, leads to hyperpolarization of the mitochondrial membrane and effectively lowers the concentration of ATP in the experimental system studied. Such changes will promote the extrusion of large amounts of hydrogen peroxide to the cytoplasm. Additionally, hyperpolarization of the mitochondrial membrane will promote penetration of the protonated form of superoxide radicals (hydroperoxyl radicals), which may explain the detection of superoxide anions with DHE in HepG2 cells after treatment with antimycin A.

Intramitochondrial superoxide anions, measured with MitoSOX, are detectable after treatment with an inhibitor concentration of at least 1 µmol/dm^3^ in the cases of both inhibitors, which confirms that these ROS do not penetrate the mitochondrial barrier due to its chemical structure and the rapidity of enzymatic reactions leading to its removal.

Changes in mitochondrial membrane potential are crucial for the transfer of reactive oxygen species from mitochondria to cytosol. There are many commercially available probes that can be used to assess degrees of mitochondrial membrane polarization, among which the most commonly used are ratiometric probes, such as JC-1 and JC-9. Unfortunately, they are often used unreflectively. As JC-1 is a ratiometric probe, the parameter corresponding to the change in mitochondrial membrane potential is the ratio of fluorescence intensity at two emission wavelengths rather than fluorescence intensity itself. First, these probes were utilized to assess the state of mitochondria with microscopic techniques [87] and only later were they adapted for flow cytometry and microplate reader-based measurements. The interpretation of data from such measurements involves difficulties that are related to the overlap of the emission of JC-1 monomers and dimers. However, the use of substances such as oligomycin and CCCP (compounds that cause mitochondrial membrane hyperpolarization and depolarization, respectively [88]) may help with data interpretation.

Taking into consideration all the studied parameters, we postulate that it possible to reliably assess the influence of virtually any substance on the production of ROS without the need for mitochondria isolation. However, the measurements should not be limited to single concentrations of xenobiotics, nor should they be limited to the measurement of a single parameter, as the relationships in question are often not linear and do not show general responses to the tested compounds. ROS generation analysis should be supported by measurements of other parameters, such as mitochondrial membrane potential and ATP concentration, as this would allow the conduction of more credible evaluations of mitochondrial status in cellular systems.

## 4. Materials and Methods

### 4.1. Cell Cultures

The HepG2 cell line was purchased from ATCC and was cultured in DMEM (Thermo Fisher Scientific, cat. no. 10566016, Waltham, MA, USA) in a Steri-Cult incubator at 37 °C, 70% humidity and a 5% CO_2_ atmosphere. Cells were passaged every third day at a confluence level of around 70%.

### 4.2. Estimation of Cell Survival with Neutral Red

Survival of HepG2 cells in response to rotenone (Sigma-Aldrich, cat. no. R8875, St. Louis, MO, USA) and antimycin A (Sigma-Aldrich, cat. no. A8674) was performed as described previously [21]. Briefly, cells were plated onto 96-well plates (Thermo Fisher Scientific, cat. no. 167008), and after 24 h rotenone and antimycin was added to the DMEM solution. After another 24 h of incubation, the culture medium was discarded and 100 mm^3^ of Neutral Red in DMEM was applied to each well. After 4 h of incubation, the medium with Neutral Red was discarded, the cells were quickly washed 3 times with 150 mm^3^ of PBS and 100 mm^3^ of solubilizing solution was added to each well. The solubilizing solution consisted of 50% ethanol, 1% acetic acid and 49% water. Plates were shaken for 15 min in an Eppendorf Thermomixer (700 rpm), and absorbance was read at 560 nm wavelength in an EnVision 2104 Multilabel Reader.

### 4.3. Measurement of Reactive Oxygen Species and Mitochondrial Potential

Dihydrorhodamine 123, DHE, MitoSOX and CMX Rosamine were used for the determination of ROS and mitochondrial membrane potential, respectively. All measurements were conducted with an LSR II flow cytometer. HepG2 cells were seeded onto 6-well plates (Nunc, cat. no. 140675, Waltham, MA, USA) at a density of 5·× 10^5^ cells per well. After 24 h, rotenone or antimycin A were added at a range of concentrations (0.001–100 µmol/dm^3^) for 4 h of incubation, after which the media with inhibitors were discarded and cells were trypsinized for 5 min. Next, trypsin was inactivated by the addition of 800 mm^3^ of DMEM, the cells were brought to suspension by pipetting and 500 mm^3^ of cell suspension was transferred to a cytometric test tube.

Solutions of fluorescent probes at 30 µmol/dm^3^ concentrations were prepared in DMEM and added at a volume of 100 mm^3^ to test tubes containing 500 mm^3^ of cell suspension to obtain a final concentration of 5 µmol/dm^3^. Readings were performed in FITC and PE-Texas Red channels, respectively, for dihydrorhodamine 123 and CMXRos, and in the PE channel for DHE and MitoSOX. For validation of the mitochondrial potential assay, CCCP was used at a final concentration of 5 μmol/dm^3^.

### 4.4. ATP Concentration Assessment

ATP concentration was measured using a commercially available kit (Thermo Fisher Scientific, cat. no. A22066), according to the manufacturer’s instructions. Readings were performed with an EnVision 2104 Multilabel Reader (PerkinElmer, Waltham, MA, USA).

## Data Availability

Data are available upon request.
